# Barriers and enablers for deprescribing among older, multimorbid patients with polypharmacy: an explorative study from Switzerland

**DOI:** 10.1186/s12875-019-0953-4

**Published:** 2019-05-14

**Authors:** Stefan Zechmann, Cosima Trueb, Fabio Valeri, Sven Streit, Oliver Senn, Stefan Neuner-Jehle

**Affiliations:** 1 Institute of Primary Care, University of Zurich, Pestalozzistrasse 24, 8091 Zurich, Switzerland; 2 Institute of Primary Health Care (BIHAM), University of Bern, Mittelstrasse 43, 3012 Bern, Switzerland

**Keywords:** Conservatism, The burden of treatment, Devaluation, Trust, And relationship, Patient involvement

## Abstract

**Background:**

Polypharmacy is an increasing problem, leading to increased morbidity and mortality, especially in older, multimorbid patients. Consequently, there is a need for reduction of polypharmacy. The aim of this study was to explore attitudes, beliefs, and concerns towards deprescribing among older, multimorbid patients with polypharmacy who chose not to pursue at least one of their GP’s offers to deprescribe.

**Methods:**

Exploratory study using telephone interviews among patients of a cluster-randomized study in Northern Switzerland. The interview included a qualitative part consisting of questions in five pre-defined key areas of attitudes, beliefs, and concerns about deprescribing and an open explorative question. The quantitative part consisted of a rating of pre-defined statements in these areas.

**Results:**

Twenty-two of 87 older, multimorbid patients with polypharmacy, to whom their GP offered a drug change, did not pursue all offers. Nineteen of these 22 were interviewed by telephone. The 19 patients were on average 76.9 (SD 10.0) years old, 74% female, and took 8.9 (SD 2.6) drugs per day. Drugs for acid-related disorders, analgesics and anti-inflammatory drugs were the three most common drug groups where patient involvement and the shared-decision-making (SDM) process led to the joint decision to not pursue the GPs offer. Eighteen of 19 patients fully trusted their GP, 17 of 19 participated in SDM even before this study and 8 of 19 perceived polypharmacy as a substantial burden. Conservatism/inertia and fragmented medical care were the main barriers towards deprescribing. No patient felt devalued as a consequence of the deprescribing offer. Our exploratory findings were supported by patients’ ratings of predefined statements.

**Conclusion:**

We identified patient involvement in deprescribing and coordination of care as key issues for deprescribing among older multimorbid patients with polypharmacy. GPs concerns regarding patients’ devaluation should not prevent them from actively discussing the reduction of drugs.

**Trial registration:**

ISRCTN16560559.

**Electronic supplementary material:**

The online version of this article (10.1186/s12875-019-0953-4) contains supplementary material, which is available to authorized users.

## Background

Polypharmacy (i.e. taking > 5 drugs/day) is an increasing problem for multimorbid patients, in particular, older individuals [1–4]. If the drugs are used inappropriately, i.e. with an inadequate ratio of benefit and harm or not meeting patients’ needs [5, 6], polypharmacy leads to increased morbidity, hospital admissions [7–9], health-related costs [10] and mortality [5, 6, 11]. In Switzerland, 21% of all patients with polypharmacy were additionally affected by potentially inappropriate medication according to lists by Beers and Priscus [12–14]. Consequently, there is an ongoing call for deprescribing and several approaches to pursue have been developed [15–21]. Approaches included lists and criteria e.g. STOP/START criteria or the EURO-FORTA (Fit fOR The Aged) list as well as electronic Decision Support (PRIMA-eDS) systems [20–22]. Despite encouraging results concerning the reduction of drugs, findings concerning the impact of deprescribing on clinical outcomes are variable. Especially implementation of deprescribing into clinical practice remains a major challenge. Attitudes, beliefs, and concerns resulting in individual barriers both on patients as well as general practitioners (GPs) side have a major impact on how patients respond to initiatives to deprescribe. [23–29]. For a successful implementation, barriers such as understanding (in) appropriateness of a drug, of the deprescribing process or patient’s fear of withdrawal have to be taken into account [26]. So far studies explored these barriers either among patients not affected by deprescribing, while studies among patients who actually chose not to pursue an offer to deprescribe do not exist.

Thus the aim of our study was to explore barriers towards deprescribing among older, multimorbid patients with polypharmacy in Switzerland who did not pursue their GPs offer. The results may help to optimize future deprescribing initiatives.

## Methods

### Context and study setting

For this explorative study, we analyzed data from a cluster-randomized study including 334 multimorbid patients (inclusion criteria: ≥ 60 years old, taking ≥5 drugs per day) recruited by GPs in Northern Switzerland. This study investigated the long-term effect of an algorithm to reduce medication in comparison to usual care [16]. The algorithm included four questions the GP should apply for each and every drug his patient received. Based on this questions the GP had offer four alternatives for each drug. Either to stop a drug, adjust its dosing or substitute with an alternative or leave it unchanged. In a SDM process the GP and the patient then decided whether to pursue the GP’s offer or not. Details see Additional file 1 for the algorithm used and Additional file 2 for actual case report form (CRF) used.

For the reporting, consolidated criteria for reporting qualitative research (COREQ-reporting quidelines) were used as appropriate [30], see details Additional file 3.

### Definitions and study sample

**Fig. 1:**
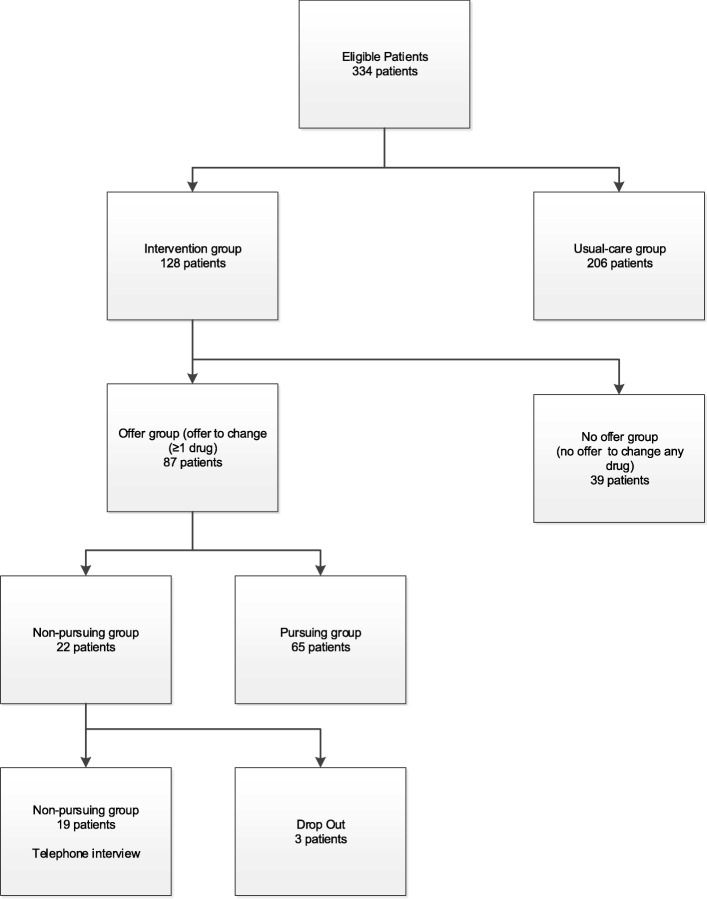
Inclusion flowchart: Of the 334 patients included in the original cluster-randomized study 19 were finally interviewed

We classified *intervention group* patients (patients exposed to the deprescribing algorithm [16] as outlined in Fig. 1 into

- *offer group*: patients having received an offer from their GP to change at least one of their drugs
- *no-offer group*: patients having received no offer from their GP to change any of their drugs

Patients in the *offer group* were further classified into

- *pursuing group:* patients pursuing their GP’s offer
- *non-pursuing group*: patients choosing not pursue at least one of their GP’s offers

Patients were eligible for the telephone interview if classified in the “no pursue group”. Patients consent for a potential telephone interview was obtained at baseline of the main study, as well as patients’ characteristics [16]. Qualitative and quantitative data were collected 1 month after the final consultation, i.e. 13 months after patient’s study entry, by semi-structured telephone interviews. This time point was chosen to maintain blinding during the main study’s follow-up of 12 months.

### Interview guide development

Before conducting the interviews, we constructed an interview guide as follows: First, we searched for factors known to impact the deprescribing process in the literature [23–26, 29, 31–38] and summarized, simplified, and adapted these findings and finally defined five key areas, based on clinical relevance, by consensus within our study group. The five key areas were trust/relationship between patient and GP, involvement of the patient, conservatism/inertia, burden of treatment and devaluation. For each key area, we developed one or two questions to be rated by the patient in regard to relevance for their decision. An additional file shows the actual interview guide used for this study (Additional file 2). We also added an open exploratory question in order to provide room for any new insights from patients’ answers, related to key areas and corresponding questions for the interview guide.

Finally, we tested the interview guide’s key areas and the corresponding questions/statements in pilot interviews until no more additional insights or new understandings occurred. First, the interview guide was tested among two members of institute staff not participating in this study and secondly on two randomly assigned patients from the main study. The latter data was not used for this study.

### Conduction of interviews and data collection

Between November 2016 and January 2017 a member of the study group (SZ), already involved in the main study, conducted one-to-one telephone interviews with patients in their homes, using the guide. SZ asked if they were alone in the room and then introduced himself at the beginning of the telephone contact and told each patient his working-position, explained reasons for this research and reminded them of their participation in the main study that ended a month ago. Additionally he asked them to re-confirm their participation in the telephone interview given initially at main study entry. In case of non-response, telephone calls were repeated until the patient was reached or reason for non-response was clarified. Interviews lasted approximately 15 min, were first transcribed verbatim on paper (ethics permission for audio-recording was not sought for) and later transferred into the electronic database. No repeat interviews were carried out nor were transcripts returned to interviewees for correction or feedback on findings.

### Data analysis

For content-analysis we used a thematic multi-stage procedure [36, 39–44]: First two researchers (SZ, CT) independently summarized and coded patients’ answers until saturation of codes was reached (Saturation was defined as no new codes emerging any more in the course of analyzing the interview transcripts). In a second step, we classified the previously coded answers into five predefined key areas. In a third step, each researcher suggested potential new key areas for these answers not fitting into the predefined areas. In a fourth step, they checked for internal agreement and discussed coding and potential new key areas The results were then discussed within the entire study group and a decision on new key areas was made. For the purpose of reporting data, we post-hoc decided to collapse answers of different questions once we realized patients gave answers fitting key areas not specifically asked for in the according question. Proportions derived from coding of patient answers, taking into consideration all answers, independent of the fact whether the answer was consistent with the specific question or not. For example the meaning of 18/19 in the case of trust and relationship implies that 18 out of 19 patients in this study had given at least one answer that was coded into this key area by our researchers. We also added a P for the patient and a number to allow alignment of quotes to the individual patient.

We measured the frequencies of positive or negative answers to questions corresponding with the key areas and used a 5-point Likert scale (except question number 7 where we used a binary answer) for the statement ratings. For descriptive analysis, we used numbers and percentages for categorical variables (Likert scale) and means and standard deviation (SD) for continuous variables (patients’ characteristics). Data management and all calculations were performed with database program SecuTrial®, the database program SQL®, and the statistics program R® [45–47].

## Results

Twenty-two (25.3%) of 87 patients receiving an offer to change drugs chose not to pursue at least one of their GPs’ offers. Three out of these 22 dropped out due to death (two patients) or cognitive decline (one patient) making telephone interviews impossible. The mean age of the remaining 19 patients, all of which agreed to participate was 76.9 (SD 10.0) years old, 12/19 (73.7%) were female (details see Fig. 1 and Table 1). They took a mean of 8.9 (SD 2.6) drugs and received offers to change for 68 drugs. Following the SDM-process the joint decision between GP and patient led to pursue 34 (50%) of these offers while the other half was not pursued (like dose changes). For details on drug groups patients actually chose not to pursue their GPs’ offer see Additional file 4.

**Table 1:** Patient characteristics, in brackets unity of individual characteristics

Characteristics	
Patients (n)	19
Age (mean/SD) [years]	76.9 (10.0)
Female (n/%)	14 (73.7)
Weight (mean/SD) [kg]	76.5 (20.2)
Blood pressure (mean/SD) [mmHg]	
Systolic	127.7 (8.3)
Diastolic	76.2 (11.2)
Hba1c (mean/SD) [%]	7.4 (0.6)
Drugs (mean/SD)	8.9 (2.6)
Quality of life (mean/SD) [0–100]	65.2 (17.1)
Severity of chief complaint (mean/SD) [0–10]	5.3 (2.4)
Living situation (n/%)	
Living alone	4 (21.1)
Living with family	10 (52.6)
Living in a care center	5 (26.3)
Length of patient-GP relationship (mean/SD) [years]	10.4 (8.8)
Drugs not changed while recommended(n)	34
Drugs for acid related disorders (n/%)	8 (23.5)
Analgetics (n/%)	4 (11.2)
Anti-inflammatory and antirheumatics (n/%)	4 (11.2)
Psycholeptics (n/%)	3 (8.8)
Psychoanalgetics (n/%)	3 (8.8)

The content of the squared bracket specifies the unit used for the specific characteristic

Table 1 According to data obtained from a cluster-randomized study [16]. Quality of life: actual health status using a Visual Analog Scale (VAS) ranging from 0 (worst imaginable) to 100 (best imaginable), Severity of complaint using a VAS ranging from 0 (no complaint at all) to 10 (unbearable). Drug pharmaceutical groups ranked by frequency and restricted to the five most frequent groups.

### Key areas

The five predefined key areas were trust/relationship between patient and GP, the involvement of the patient, conservatism/inertia, burden of treatment and devaluation. The following quotes provide more insight into these areas.

### Trust and relationship

18/19 patients reported to fully trust their GP.

*“I fully trust my GP, he recommends what’s best for me.” P1*

*“My GP is the best possible placebo, talking to him helps me a lot.” P6*

*“He knows what’s important to me, he cares. He sees more than the costs and effectiveness of drugs.” P8*

*“I would change my GP in case of lacking trust.” P11*

*“My GP discusses my medication with my caregivers, I fully trust them.” P14*

### Involvement

17/19 patients either had previous consultations focusing on medication or wished for it. 10/19 patients had no medication list. Six of these 10 patients wished for it.

*“My GP informs me well, finally it is his turn to make a decision. I do not understand it anyway”. P1*

*“I know my medication, but what happens in case of an emergency? In that case, a medication list would be perfect.” P3*

*“We discuss my medication-scheme regularly. My GP knows what’s recommended, but in the end, I am the only one who knows what’s good for me.” P9*

### Conservatism/inertia

15/19 patients felt that all of their drugs were necessary or beneficial for their daily living. 9/19 mentioned the feeling of security entailed with their drugs. 6/19 patients felt deprescribing actually took away something which had been beneficial for them in the past. None out of 19 patients did regret their decision not to pursue their GP’s offer.

*“I would love to reduce medication. Nevertheless, I need the drugs my GP wanted to stop.” P3*

*“My answer is generally NO to changes in the first place. I take new drugs only after reviewing information and evaluation of non-drug alternatives.” P5*

*“I’d generally love to stop medication! But I need the painkiller and proton pump inhibitor.” P8*

*“I have good experience with my drugs up to now, so better not change the winning team.” P12*

*“I take my anti-acidity drug for almost 40 years – got perfectly used to it.” P13*

*“I feel well now, obviously all of my drugs are needed.” P15*

### Burden of treatment

8/19 patients perceived that they were taking too many drugs.

*“There have been so many drug changes that I lost track of the number. Anyway, the pharmacy packs them daily.” P1*

*“First I didn’t like the fact, but now I accepted that I need them.” P16*

### Devaluation

None out of 19 patients reported a feeling of worthlessness as a consequence of deprescribing offer. (Only one patient reported that he had “somewhat” the feeling of worthlessness, but not “to a great extend”).

### New key area

6/19 patients mentioned as an answer in the additional open question that the involvement of too many different doctors was a problem for them. According to the procedure described in the method section, we defined this topic as a new key area and labeled it as *“fragmented medical care”* i.e. several caregivers are in charge for the patient.

*“Each and every doctor takes care of their medication - almost one specialist for every drug.” P4*

*“I have got my GP for “real” medication and the external team for alternative medication.” P16*

### Rating frequencies

**Fig. 2:**
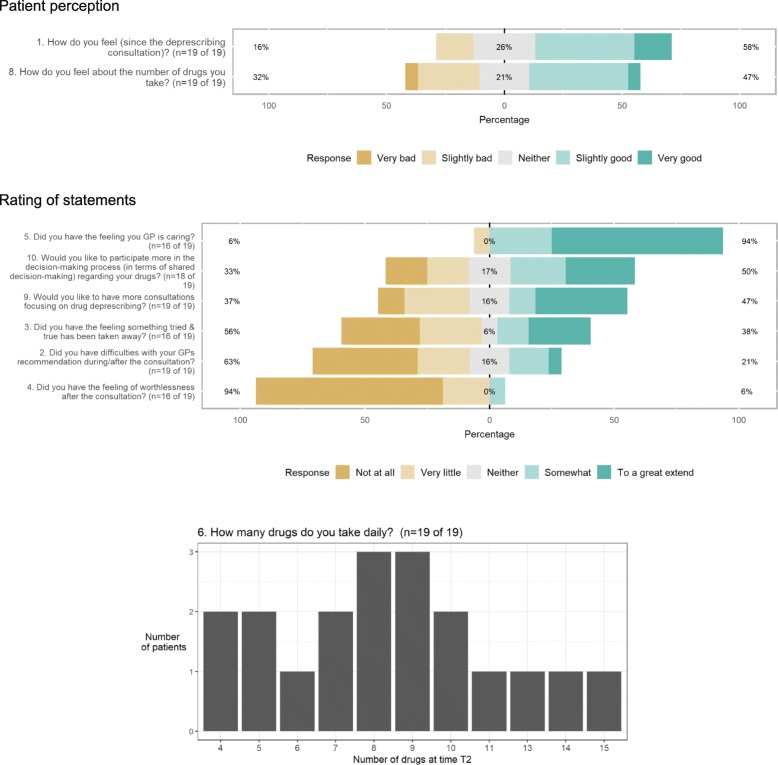
Patients’ perception and rating of statements shows patients’ results rated on a 5-point Likert scale ranked according to patients’ perception as in question 1 and 8: from 1 = “Very bad” to 5 = “Very good”, and according to patients` rating of statements as in statement 2–5 and 9–10: from 1 = “Not at all” to 5 = “To a great extend”. Question 6 shows the number of patients stratified by their number of drugs taken daily. Number 7 is not shown. The number at the beginning of each question respectively statement equals their number in the interview guide (Additional file 2)

Patients’ perception and rating for the predefined statements are shown in Fig. 2. The statement number equals the number in the interview guide. See (Additional file 2). Patients strongly approved that they had the feeling their GP was caring (statements no. 5) and moderately approved that they would like to participate more in the decision-making process regarding drugs and would like to have more consultations focusing on deprescribing (statements no. 10 and no. 9) with approval rates of 94, 50, and 47% respectively. Patients rather disapproved that they had the feeling something tried and true had been taken away or they had a feeling of worthlessness after the consultation (statement no. 3 and 4) with disapproval rates of 56 and 94%, respectively). Only one patient (6%) rather approved to the feeling of worthlessness after the consultation, while the other 15 (94%) disapproved this feeling. 9 out of 19 patients had a medication list, while the other 10 without list declared that they would be interested in having one (question 7).

## Discussion

When GPs offered to deprescribe, 22 of 87 of their older, multimorbid patients chose not to pursue at least one of their GPs offers following a SDM process. In this study, we were able to interview 19 of those 22 patients. They chose not to pursue to deprescribe, although a majority of them fully trusted their GP and was already involved in SDM before the study. About one third perceived polypharmacy as a substantial burden. We found the following reasons for patients’ decision not to pursue their GPs offer: 1) patients thought that they needed each drug; 2) patients felt deprescribing would remove something beneficial from them, and 3) too many physicians were involved in medication management. Contrary to what was expected from literature [18], no patient felt devalued as a consequence of the deprescribing offer. The main findings in the key areas trust and devaluation were supported by patients’ ratings of predefined questionnaire statements, while other key areas such as burden of treatment have been approved to a lower degree in these ratings.

*Trust* is of utmost importance for a good patient-GP relationship [33, 48], a prerequisite for a SDM process and therefore most relevant for deprescribing procedures [49]. In our study, patients stated a high level of trust and a feeling that their GP cared a lot. This is reflected in a long average duration of patient-physician-relationship as well. Palagyi et al. reported that the willingness to change a drug was strongly dependent on the GP as a central and trusted person [33]. Nevertheless, high levels of trust did not encourage patients to accept all of their GPs’ offers to change drugs in our study. Trust could even be an enabler for choosing not to pursue the offer, as patients may feel confident that their GPs are open-minded for listening to their opinions and concerns. Thus, patients with a high level of trust in their GPs even may feel encouraged *not* to pursue the offer. A decision not to pursue the GP’s offer, is meeting the spirit of SDM and therefore is a positive outcome [50].

The inconsistency between willingness to reduce drugs combined with great trust in the GP on the one side and the decision not to pursue the offer to do so on the other side has been shown in literature before, although not on such a large extent [51].

The lack of *patient involvement* and a low degree of SDM were important barriers against deprescribing in the review by Anderson et al. [24]. In our study sample, all patients were involved in a SDM process due to the type of study intervention and the majority reported to have participated in a SDM process before the study and were keen to participate again in the future. We conclude that a high level of patient involvement and SDM does *not* necessarily mean that patients will pursue deprescribing when offered by the GP. Similar to the ambiguous role of trust, high levels of patient involvement and SDM may empower patients to take a decision along their own attitudes and concerns, which might not be in line with their GP’s offer. Although potentially hindering deprescribing, they are most valuable for a patient-centered medicine. Thus the joint decision between GP and informed patient to continue a medication rather than pursuing an offer for deprescribing could be the best decision for the patient if in line with her/his values and preferences.

*Conservatism and inertia* were other important barriers towards deprescribing mentioned by our patients, as previously reported [24]. Potential reasons are the fear of losing a beneficial drug effect, the fear of withdrawal effects, as well as non-specific fears [24, 26, 51–53]. In our study, the potential loss of a beneficial drug effect matched with patients’ perception that they needed their drugs for symptom relief. A closer look at the drug groups where changes were not pursued showed that the majority of these drugs had rather symptomatic than prognostic effects (e.g. drugs for acid-related disorders, analgesics or anti-inflammatory /antirheumatics). The absence of symptoms at present may leave patients unclear whether this is indicating that a drug is no longer needed, or a sign that the drug is successful, and therefore necessary [54, 55]. Thus, patients may keep a drug rather than “taking risks” of a poor symptom control by stopping. This fear of symptom increase may even outweigh the perceived *burden of treatment* reported by almost half of our patients. This concern of patients may be best discussed openly during the deprescribing encounter. Krol et al. reported a 24% reduction of proton pump inhibitors by critically addressing indication and symptom relief [31]. Another approach may be communicating to patients that continuing a drug may be the greater risk than stopping it, due to interactions or side effects. For this approach, physicians have to be careful not to worry patients by this information.

*Fragemented medical care* as a barrier against deprescribing was mentioned by almost a third of our patients. This topic was reported in previous studies mainly investigating GPs’ views, but not by patients until now [25, 56, 57]. Patients feel uneasy to change a drug if it has been prescribed by another health care provider, which may be a kind of loyalty to this person. This barrier could possibly be avoided by the coordination of care, for example by a managed care approach as previously shown for potentially inappropriate medications [10]. Other approaches include computer-assisted information flow, access to expert advice, access to non-pharmaceutical options, and enhanced communication between specialists and GPs [24].

Surprisingly *devaluation* was mentioned in the statement ratings by one patient only, and never spontaneously. Previous studies reported that GPs are concerned that patients may interpret deprescribing as a sign of being given up on, similar to difficult discussions on life expectancy [18, 24, 25, 56, 58]. As a conclusion from this finding, GPs concerns regarding patients’ devaluation should not prevent them from actively discussing the reduction of drugs.

Further barriers to deprescribing discussed previously were poor insight or inertia of GPs, a low self-efficacy to address the topic with patients and feasibility issues like time constraints. Only lack of time (3/19) and missing information on possible medication or non-medication alternatives (2/19) was reported by a minority of our patients. Contrasting literature, *cost issues* or beliefs about the *appropriateness of a drug* were never mentioned by our patients [23, 34, 59, 60]. This might be owed to the Swiss health care system where the majority of costs are covered by insurance plans. Appropriateness might not be an issue for these patients as they trust their GPs and therefore did not question the rationale for their drugs.

Concerning enablers, the literature suggests the provision of enough time dedicated to deprescribing, a clear step by step plan how to change drugs and the option to restart the drug whenever necessary or required by the patient [24, 26, 61]. Major influencers, potentially enabling as well as hindering the acceptance of deprescribing among patients, are family members, peers, and media, as well as the former experience of the patient with the drug under discussion [16].

Although only half of our patients remembered to receive a medication list a majority of them declared a wish for it. This opens the room for further GPs activities to optimize future pre- and deprescribing.

### Strengths

To our knowledge, this is the first qualitative study among older, multimorbid patients with polypharmacy who actively chose not to pursue the offer of their GP to deprescribe. By this selection of patients who were exposed to a deprescribing offer of their GP *and* had experienced an own negative reaction to this offer, we had the possibility to explore attitudes and barriers towards deprescribing in a unique real-life setting. Until now, studies investigated GPs or patients views (or care-givers views) concerning attitudes and barriers against deprescribing rather in a hypothetical way. We managed to interview the vast majority (> 85%) of these specific patients. By using an explorative (qualitative and quantitative) approach were able to explore the complexity of the patient’s experience of deprescribing from several perspectives.

### Limitations

There are a number of limitations that should be considered when interpreting the results of our study.

First, there might be the recall bias due to the long latency between the initial consultation and the interview (13 months).

Second, there is a relatively small sample size of 19 patients, limiting the generalizability of our results [62]. Nevertheless, we think that for this specific setting of a consultation dedicated to deprescribing the results of this highly selected study population are of interest.

Third, although we conducted pilot interviews with our interview guide, it was not systematically validated in a separate study, thus limiting its applicability in further studies.

Fourth, we are aware of the potential bias caused by the fact that patients included in this study were previously selected to take part in a 12-months trial. A selection bias towards a generally fitter and better educated population compared to patients not invited or declining participation cannot be excluded. When interpreting findings from this study one should keep in mind that all clinical interactions between patients and GPs took place within the larger context of a clinical trial.

## Conclusion

We identified patient involvement in deprescribing and coordination of care as key issues for deprescribing among older multimorbid patients with polypharmacy. GPs concerns regarding patients’ devaluation should not prevent them from actively discussing the reduction of drugs.

## Additional files

Additional file 1: Algorithm. This file shows the algorithm used in the main study. (GIF 21 kb)

Additional file 2: Interview guide**.** This file shows the interview guide design and used in this study. (PDF 101 kb)

Additional file 3: COREQ list [30]**.** This file shows the answered COREQ list. (PDF 65 kb)

Additional file 4: Drug list. This file shows drugs were patients chose not to implement their GPs’ recommended changes (in order of frequency by indication group based on WHO ATC coding) [63]. (PDF 55 kb)

## Abbreviations

CRF: Case report form
CT: Cosima Trueb
eDS: Electronic Decision Support
Fit fOR The Aged: FORTA
FV: Fabio Valeri
GPs: General practitioners
OS: Oliver Senn
rPATD: Revised Patients’ Attitudes Towards Deprescribing
SD: Standard deviation
SDM: Shared-decision making
SNJ: Stefan Neuner-Jehle
SS: Sven Streit
SZ: Stefan Zechmann
VAS: Visual Analog Scale
