# Supplementary figures and images for: Barriers and enablers for deprescribing among older, multimorbid patients with polypharmacy: an explorative study from Switzerland
Source: BMC Fam Pract. 2019 May 14;20:64. doi: 10.1186/s12875-019-0953-4 (PMC6518702; doi:10.1186/s12875-019-0953-4)

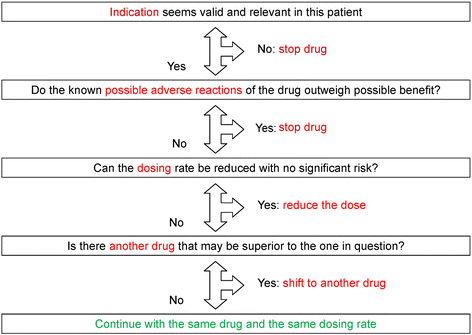

Supplement: Supplementary file 1 — Algorithm. This file shows the algorithm used in the main study. (GIF 21 kb) [file 12875_2019_953_MOESM1_ESM.gif]
