# Supplementary material for: Barriers and enablers for deprescribing among older, multimorbid patients with polypharmacy: an explorative study from Switzerland
Source: BMC Fam Pract. 2019 May 14;20:64. doi: 10.1186/s12875-019-0953-4 (PMC6518702; doi:10.1186/s12875-019-0953-4)
Supplement: Supplementary file 2 — Interview guide. This file shows the interview guide design and used in this study. (PDF 101 kb) [file 12875_2019_953_MOESM2_ESM.pdf]

Universität Zürich[UZH]

Institut für Hausarztmedizin

Chronic Care Studie

Guide Tel. Interview with patients with disorder in T0 (13 months after the first interview T0)

Centre-ID: **Pat-ID:** **Date T0:**

Patient name: **Date of birth:** **Tel:**

Date of telephone interview:

(Lead questions/narrative request; check/what has been answered, ask for concrete questions, maintenance-/control questions)

- Hello Mrs./Mr. …………………………………………., my name is Dr. Zechmann, I work at the University of Zurich. You took part in a study on medication adjustment with your GP... last year and I would like to ask you some questions about it. Do you have 10 minutes or would you prefer to make an appointment?

  ……………………………………………………………………………………………………….

- Thank you very much for taking part in our Chronic Care study. Do you know what study I am talking about?

- NO (Help out = You were chosen by your GP because you had ...... medicines and he/she has discussed all her medicines with you, suggested changes and discussed with you how to proceed "best".

- Your GP at that time has specifically proposed to change/stop:

  ……………………………………………………………………………………………………….
  ……………………………………………………………………………………………………….
  ……………………………………………………………………………………………………….

  You have suggested at the time to

  ……………………………………………………………………………………………………….
  ……………………………………………………………………………………………………….

  ……………………………………………………………………………………………………….

  Can you remember now? (Clear NO stop and say goodbye)

- YES → If it is all right for you, I will now ask you 8-9 questions about the consultation that is meanwhile over one year ago and about your medications altogether. The whole conversation will take as said about 10 min. In most of the questions there are 5 possible answers on a scale of 1-5 where 1 is the worst and 5 is the best. Should you have any questions in between please ask at any time.

1. How do you feel? (since the deprescribing consultation)?

☐(1) Very bad
☐(2) Slightly bad
☐(3) Neither
☐(4) Slightly good
☐(5) Very good

Is there a connection with the drug adjustment?
………..

………..

2. **Did you have difficulties with your GP's recommendation during / after the consultation?**

☐(1) Not at all
☐(2) Very little
☐(3) Neither
☐(4) Somewhat
☐(5) To a great extend

(Why, and if the answer is yes, which recommendations caused difficulties? Are there certain reasons?)
……

……

2.1. **"How did you experience the consultation?"**

Positive? ……………………………………………………

Negative? …………………………………………………..

Neutral? ……………………………………………………

(If negative then specifically why?)

3. **Did you have the feeling something tried & true has been taken away?**

☐(1) Not at all
☐(2) Very little
☐(3) Neither
☐(4) Somewhat
☐(5) To a great extend

(If 1 or 2, how long did you have this medicine in advance?)
……

……

4. **Did you have the feeling of worthlessness after the consultation?**
(In the sense of "It is not worth taking medication with you")

☐(1) Not at all
☐(2) Very little
☐(3) Neither

☐(4) Somewhat
☐(5) To a great extend

……

……

If positive then why?

5. **Did you have the feeling you GP is caring?**

☐(1) Not at all
☐(2) Very little
☐(3) Neither
☐(4) Somewhat
☐(5) To a great extend

……

……

6. **How many drugs do you take daily?**

☐(1) 5
☐(2) 6-7
☐(3) 8-9
☐(4) 10-11
☐(5) >=12

……

……...

7. **Did you receive a medication-list from your GP?**

☐(1) Yes
☐(2) No

If not. Would you like a list of drugs/dosage list?
……

……

8. **How do you feel about the number of drugs you take?**

☐(1) Very bad
☐(2) Slightly bad
☐(3) Neither
☐(4) Slightly good
☐(5) Very good

(Why? Experiences? Hospitalizations? Frequent changes in the past?)
……

………

9. **Would you like to have more consultations focusing on drug deprescribing?**
☐(1) Not at all
☐(2) Very little
☐(3) Neither
☐(4) Somewhat
☐(5) To a great extend

………

………

10. **Would you like to participate more in the decision-making process (in terms of shared decision-making) regarding your drugs?**

☐(1) Not at all
☐(2) To a great extend

………

………

Would you like to add something else about your use of medications in general?

………

………

Do you have any questions? Thank you very much for your precious time and cooperation.

Subjectivity/reflexivity/self-observation:
Did the conversation take place as it was planned?

☐(1) Yes
☐(2) No

………

………

Did I feel safe/unsafe?

☐(1) Yes
☐(2) No

………

…….

Did the patient feel safe/unsafe?

☐(1) Yes
☐(2) No

…….

…….

Name Interviewer:

………………………………...

Date of the interview:

………………………………...
