# Supplementary material for: Barriers and enablers for deprescribing among older, multimorbid patients with polypharmacy: an explorative study from Switzerland
Source: BMC Fam Pract. 2019 May 14;20:64. doi: 10.1186/s12875-019-0953-4 (PMC6518702; doi:10.1186/s12875-019-0953-4)
Supplement: Supplementary file 3 — COREQ list [30]. This file shows the answered COREQ list. (PDF 65 kb) [file 12875_2019_953_MOESM3_ESM.pdf]

COREQ (COnsolidated criteria for REporting Qualitative research) Checklist

Domain 1: Research team and reflexivity

Personal characteristics

1. Which author/s conducted the interview or focus group? SZ, see page 5 Conduction of interviews and data collection for questions 1 -5
2. What were the researcher’s credentials? E.g. Ph.D., MD MD
3. What was their occupation at the time of the study? Scientific researcher at the Institute of primary care, University of Zurich
4. Was the researcher male or female? Male
5. What experience or training did the researcher have? MD, scientific researcher at the Institute of Primary Care since 2015 with occasional previous scientific writing. Supported by an experienced study team of senior researchers.

Relationship with participants

6. Was a relationship established prior to study commencement? All patients included in this study were participants of a previous study conducted by parts of the study team. This previous study tested an algorithm for deprescribing in the Swiss primary care setting (1, 2), see page 5 Conduction of interviews and interviews and data collection for questions 6 and 7
7. What did the participants know about the researcher? SZ introduced himself during his first telephone contact, told the participants his position at the university and explained reasons for doing the research. He reminds them of the study they just finished about a month ago.
8. What characteristics were reported about the interviewer/facilitator? The authors reported no financial or non-financial competing interests. Looking at authors` publication lists one can clearly see their interest and expertize in the topic of primary care and polypharmacy, see page 14 Competing interest.

Domain 2: Study design

Theoretical framework

9. What methodological orientation was stated to underpin the study? For content-analysis we used a thematic multi-stage procedure based on the following (3-9). Details see page 5 and 6 Data analysis.

Participant selection

10. How were the participants selected? A specific subgroup among participants of a previous study, based on study criteria. Details see page 3 and 4 Context and study setting and Definitions and study sample.
11. How were the participants approached? Telephone, see page 5 Conduction of interviews and data collection
12. How many participants were in the study? The main study (2) included 334 participants. For this study 22 participants were eligible and 19 consented in this telephone interview. All participants consented to potentially take part in this study when they consented to take part in the main study. Details see figure 1. and page 3 and 4 Context and study setting.
13. How many people refused to participate or dropped out? Reasons?

Three out of 22 eligible participants dropped out. Two participants dropped out due to death and one participant due to cognitive decline making participation in the telephone interviews impossible. Details see page 6 Results.

Setting

14. Where was the data collected? Patients' baseline characteristics were obtained from the main study (2). Qualitative and quantitative data were collected one month after the final consultation, i.e. 13 months patient's study entry, by semi-structured telephone interviews. Details see page 3 and 4 Context and study setting
15. Was anyone else present besides the participants and researchers? SZ asked if the patients were alone. All of them answered to be by themselves at the time of the telephone interview, see Methods section, Conduction of interviews and interviews and data collection.What are the important characteristics of the sample? Participants of a cluster-randomized study including multimorbid patients (inclusion criteria: ≥ 60 years old, taking ≥ 5 drugs per day) recruited by general practitioners (GPs) in Northern Switzerland. Details see page 5 Context and study setting.

Data collection

16. Were questions, prompts, guides provided by the authors? Was it pilot tested? An interview guide was developed and pilot-tested. Details see page 4 and 5 Interview guide development
17. Were repeat interviews carried out? If yes, how many? No, see page 5 Conduction of interviews and data collection for question 17 - 20
18. Did the research use the audio or visual recording to collect the data? No
19. Were field notes made during and/or after the interview or focus group? Yes, answers were manually documented on paper-form while the telephone interview was conducted.
20. What was the duration of the interviews or focus group? Approximately 10-15min, the exact duration was not documented and dependent on the patient's ability to answer SZs questions and their will to report further.
21. Was data saturation discussed? Yes. Details see page 5 Data analysis.
22. Were transcripts returned to the participant for comments and or correction? No, see page 5 Conduction of interviews and data collection

Domain 3: analysis and findings

Data analysis

6. How many data coders coded the data? Two researchers (SZ, CT) independently summarized and coded patients` answers, see Methods section, Data analysis
7. Did the authors provide a description of the coding tree? No.
8. Were themes identified in advance or derived from the data? Both, 5 key areas were identified in advance, while 1 newly derived from interview data. Details see page 4 and 5 Interview guide development and page 6 Results.
9. What software, if applicable, was used to manage the data? Patients characteristics including medication and SDM decision available from the main study were managed using secutrial® SQL® and R®. Details see page 5 Data analysis.
10. Did participants provide feedback on the findings? No, see page 5 Conduction of interviews and data collection

Reporting

29. Were participant quotations presented to illustrate the themes/findings? Yes. Each quotation can be allocated to the source=patient by its added number, but is presented in an anonymous way. See page 6 Results for questions 29 – 32.
30. Was there consistency between the data presented and the findings? Yes.
31. Were major themes clearly presented in the findings? Yes.
32. Is there a description of diverse cases or discussion of minor themes? Yes.

References

1. Neuner-Jehle S, Krones T, Senn O. [Systematic elimination of prescribed medicines is acceptable and feasible among polymorbid family medicine patients]. Praxis. 2014;103(6):317-22.
2. Hasler S, Senn O, Rosemann T, Neuner-Jehle S. Effect of a patient-centered drug review on polypharmacy in primary care patients: study protocol for a cluster-randomized controlled trial. Trials. 2015;16:380.
3. Korstjens I, Moser A. Series: Practical guidance to qualitative research. Part 4: Trustworthiness and publishing. The European journal of general practice. 2017:1-5.
4. Moser A, Korstjens I. Series: Practical guidance to qualitative research. Part 3: Sampling, data collection and analysis. The European journal of general practice. 2018;24(1):9-18.
5. Mayring P. Qualitative inhaltsanalyse. Handbuch qualitative Forschung in der Psychologie: Springer; 2010. p. 601-13.
6. Braun V CV. Using thematic analysis in psychology. Qual Res Psychol. 2006;;3(2)::77-101.
7. Geense WW, van de Glind IM, Visscher TL, van Achterberg T. Barriers, facilitators and attitudes influencing health promotion activities in general practice: an explorative pilot study. BMC family practice. 2013;14:20.
8. Hardy V, Thompson M, Alto W, Keppel GA, Hornecker J, Linares A, et al. Exploring the barriers and facilitators to use of point of care tests in family medicine clinics in the United States. BMC family practice. 2016;17(1):149.
9. Schroder L, Flagel K, Goetz K, Steinhauser J. Mobility concepts and access to health care in a rural district in Germany: a mixed methods approach. BMC family practice. 2018;19(1):47.
