# Supplementary material for: Barriers and enablers for deprescribing among older, multimorbid patients with polypharmacy: an explorative study from Switzerland
Source: BMC Fam Pract. 2019 May 14;20:64. doi: 10.1186/s12875-019-0953-4 (PMC6518702; doi:10.1186/s12875-019-0953-4)
Supplement: Supplementary file 4 — Drug list. This file shows drugs were patients chose not to implement their GPs’ recommended changes (in order of frequency by indication group based on WHO ATC coding) [63]. (PDF 55 kb) [file 12875_2019_953_MOESM4_ESM.pdf]

Drug list

Drugs were patients chose not to implement their GPs`recommendation for change by order of frequency by indication group based on WHO- ATC coding (1).

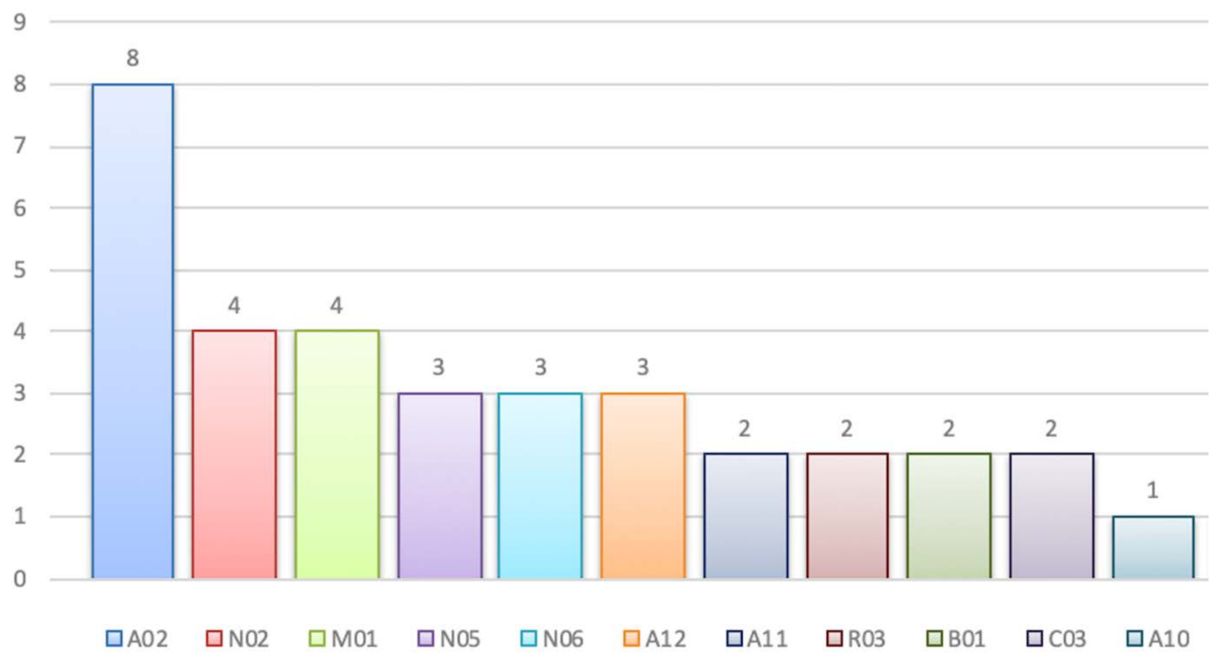

A02 **DRUGS FOR ACID RELATED DISORDERS** **(8)**

N02 **ANALGESICS** **(4)**

M01 **ANTIINFLAMMATORY AND ANTIRHEUMATIC PRODUCTS** **(4)**

N05 **PSYCHOLEPTICS** **(3)**

N06 **PSYCHOANALEPTICS** **(3)**

A12 **MINERAL SUPPLEMENTS** **(3)**

A11 **VITAMINS** **(2)**

R03 **DRUGS FOR OBSTRUCTIVE AIRWAY DISEASES** **(2)**

B01 **ANTITHROMBOTIC AGENTS** **(2)**

C03 **DIURETICS** **(2)**

A10 **DRUGS USED IN DIABETES** **(1)**

1. WHO ATC Coding. URL: https://www.whocc.no/, Accessed 14.01.2019
